# Effects of Different Stocking Densities on Snail *Bellamya purificata* Foot Muscle Nutritional Quality and Metabolic Function

**DOI:** 10.3390/ani14243618

**Published:** 2024-12-15

**Authors:** Yingyue Lou, Rui Jia, Bing Li, Linjun Zhou, Jian Zhu, Yiran Hou

**Affiliations:** 1 Wuxi Fisheries College, Nanjing Agricultural University, Wuxi 214081, China; louyingyue@hotmail.com (Y.L.); jiar@ffrc.cn (R.J.); lib@ffrc.cn (B.L.); 2 Key Laboratory of Integrated Rice-Fish Farming Ecology, Ministry of Agriculture and Rural Affairs, Freshwater Fisheries Research Center, Chinese Academy of Fishery Sciences, Wuxi 214081, China; zhoulinjun@ffrc.cn

**Keywords:** *Bellamya purificata*, foot muscle nutritional quality, stocking density, transcriptome, metabolomics

## Abstract

Snail *Bellamya purificata* is not only useful for bioremediation, purifying aquaculture environments, but it is also a commercially valuable and nutritionally rich aquatic product. To analyze the effect of various stocking densities on the muscle nutritional quality and metabolic functions of *B. purificata*. The transcriptome and metabolome were analyzed and set up three different density groups—low (LD, 234.38 g/m^2^), medium (MD, 468.75 g/m^2^), and high (HD, 937.5 g/m^2^). The results of the study showed that the weight gain (WG) and specific growth rate (SGR) of *B. purificata* in the MD and HD groups were significantly lower compared to the LD group. High stocking density significantly reduced the oleic acid (C18:1n9c), linoleic acid (C18:2n6c), alpha-linolenic acid (C18:3n3), eicosadienoic acid (C20:2), erucic acid (C22:1n9), docosahexaenoic acid (DHA, C22:6n3), and lignoceric acid (C24:0) levels within snail foot muscle. Most of the identified differentially expressed genes (DEGs) were categorized as Signal transduction, according to the Kyoto Encyclopedia of Genes and Genomes (KEGG); these genes were categorized into Transport and catabolism, Endocrine system, and Immune system. A total of 11 upregulated DEGs and 19 downregulated DEGs were identified and confirmed to be associated with density stress. The identified metabolites were mainly enriched in the Metabolism category, with 620 differential metabolites identified in positive ion (POS) mode and 265 differential metabolites identified in the negative ion (NEG) mode among different stocking density groups. The differential metabolites affected by stocking density were primarily amino acids, nucleic acids, vitamins, and lipid metabolites. There were 8 upregulated differential metabolites and 14 downregulated differential metabolites identified and confirmed to be associated with density stress. These findings elucidated the response mechanisms of *B. purificata* to adverse stocking density conditions and provide data and a theoretical basis for selecting appropriate stocking densities for *B. purificata*.

## Simple Summary

Snail *Bellamya purificata* not only improves resource efficiency and purifies the aquaculture environment but is also a commercially valuable and nutritious aquatic product. Stocking density is a key determinant of aquaculture yield; sustained high densities cause stress and competition for resources among species, negatively impacting their growth, survival, and quality. This, in turn, reduces the economic benefits of aquaculture. However, research on the impact of stocking density on *B. purificata* remains limited. The aim of this study was to study the influence of different stocking density on the nutritional quality and metabolic function of snail *B. purificata* foot muscle through transcriptomic and metabolomic analysis. The findings indicated that high-density conditions adversely affect the nutritional quality and metabolic function of the snail foot muscle. These findings give guidance for selecting appropriate stocking densities for *B. purificata*.

## 1. Introduction

The snail *Bellamya purificata*, a species from the family Viviparidae and genus *Bellamya*, thrives in freshwater habitats across Asia, playing a crucial role in wetland ecosystems [1]. Snail *B. purificata* facilitates not only bioremediation and purifies the aquaculture environment but is also a commercially valuable and nutritious aquatic product [2,3,4,5,6,7,8]. Its muscle contains a balanced amino acid profile, making it a premium protein source, especially rich in flavor-enhancing amino acids, thus, offering great potential for seasoning production and advanced processing [9]. Meanwhile, *B. purificata* is not only a crucial bait for some waterfowl but also an important part of the daily diet for many people, attracting increasing attention [10]. Owing to its large size and rapid growth, the snail *B. purificata* is the most favored and suitable species for aquaculture within the *Bellamya* genus [11]. In 2022, shellfish aquaculture in China accounted for approximately 28.5% of the national output, reaching 15.89 million tons [12]. However, despite the industry’s rapid growth, research on *B. purificata* cultivation is still relatively limited. Also, most of the research has focused on its ecological impacts, with relatively little research on the nutritional quality of its muscle.

The key factor in determining aquaculture production is stocking density, with increased density commonly adopted to boost both productivity and profitability [13]. While high-density stocking optimizes water resource utilization and enhances aquaculture income, sustained high densities can introduce stress that leads to competition for food and space among aquatic species, negatively affecting their growth, survival rates, and quality, ultimately reducing the economic returns of aquaculture [14,15,16,17,18,19,20,21]. The high-density pressure has been shown in previous studies to weaken antioxidant and immune functions in aquatic animals, while also affecting their nutritional quality and metabolic functions [22,23,24,25]. Wang et al. (2022) showed that stocking density impacted growth performance, nutrient quality, immunity and water quality of Chinese Mitten Crab in pond ecosystems [26]. Gao et al. (2023) indicated that metabolic diseases such as fatty liver and other related conditions of grass carp were induced under high-density culture mode, which reduced meat quality and consequently affecting its aquaculture efficiency [27]. However, few have explored the effect of stocking density on *B. purificata*, especially concerning the potential impacts of high-density stocking on the snail foot muscle nutritional quality and metabolic function.

This study aimed to elucidate the effects of different stocking densities on the nutritional quality and metabolic function of *B. purificata* foot muscles. In this study, we analyzed the differences in amino acids, fatty acids, transcriptomes, and metabolomes of *B. purificata* foot muscle to lay a theoretical foundation for determining the optimal stocking density for *B. purificata* culture.

## 2. Methods and Materials

### 2.1. Experimental Design and Sample Collection

Experiments were conducted at the Freshwater Fisheries Research Center (FFRC), Chinese Academy of Fishery Sciences (CAFS) (120.250479° E, 31.51581° N, Wuxi, China). According to the previous studies relevant to the snail *B. purificata* cultivation, three stocking density groups were established for snail *B. purificata*—low (LD, 234.38 g/m^2^), medium (MD, 468.75 g/m^2^), and high (HD, 937.5 g/m^2^)—with three replicates per group, using nine glass tanks (80 cm × 40 cm × 45 cm) each lined with 7 cm thick sediment layer [3,28,29]. Experimental snails *B. purificata* and associated experimental sediments were obtained from the FFRC Yixing Aquaculture Plant (119.93913° E, 31.31698° N). Prior to the experiments, the sediment was sun-dried, powdered, sifted, and mixed thoroughly through a 100-mesh sieve to ensure homogeneity and consistency. The tank was filled with filtered fresh water and the sediment was allowed to settle for 14 days. The average wet weight of snails was 2.53 ± 0.01 g. They were first domesticated in glass tanks (consistent with those used during the experiments) at a density of 234.38 g/m^2^, and then randomly placed in glass tanks at the corresponding stocking densities after 14 days. The snails were fed with a commodified feed (Zhejiang Haida Feed Co., Ltd., Shaoxing, China) at 16:00 every day at a rate of about 2% of the snails’ body weight. The fresh water used for the experiment was aerated tap water, with ammonia nitrogen at 0.09 ± 0.02 mg/L, dissolved oxygen at 6.5 ± 0.2 mg/L and pH at 7.14 ± 0.06. One-third of the water was replaced at two-day intervals. The duration of the experiment was 80 days. All snails remained healthy, and none died.

After the experiment, 10 snails were randomly selected from each glass tank and subjected to a 24 h fasting period before their foot muscle samples were collected. Samples collected from the same glass tank were combined into a single sample. To ensure the stability of the foot muscle samples, all samples were stored in a refrigerator at −80 °C.

### 2.2. Growth Parameters Analysis

Growth performance was evaluated by analyzing weight gain (WG; %), weight specific growth rate (SGR; %). The formulas are as follows:

WG = 100 × (W2 − W1)/W1

SGR = 100 × (lnW2 − lnW1)/T

where W1 and W2 are the initial body weight (g) and final body weight (g), respectively, and T is the total number of days of the test.

### 2.3. Amino Acids Determination

A total of 3 g of foot muscle tissue was weighted and put into the hydrolysis tube, followed by the addition of 15 mL of 6.0 M hydrochloric acid. The tube was immersed in coolant for five minutes and flushed with nitrogen to remove oxygen before sealing. Sample hydrolyzed for 22 h at 110 °C. Post-hydrolysis, the content was transferred to a 50 mL volumetric flask and filled to the mark with deionized water. A 1.0 mL sample of the filtrate was vacuum dried at 40 °C. The residue was re-dissolved in 1.0 mL of sodium citrate buffer at pH 2.2, filtered through a 0.22 μm filter, and the resulting supernatant was analyzed using a Hitachi LA8080 amino acid analyzer (Hitachi, Kyoto, Japan).

### 2.4. Fatty Acids Determination

Approximately 0.5 g of dry muscle tissue were weighted and hydrolyzed with 8.3 M hydrochloric acid at 70–80 °C for 40 min. Subsequently, the total lipid was extracted from the hydrolysate by a diethyl ether and petroleum ether mixture (1:1, *v*/*v*) complying with Chinese national standards (GB5009.168-2016) [30]. Next, 8 mL of 2% sodium hydroxide methanol solution was added to the extracted lipids, and the mixture was connected to a reflux condenser and refluxed in a water bath maintained at 80 °C ± 1 °C until the oil droplets had dissipated. Then, 7 mL of 15% boron trifluoride methanol solution was introduced through the upper end of the condenser, and the reflux was continued for an additional 2 min at the same temperature. The condenser was rinsed with a small volume of water to remove any residues. Upon cessation of heating, the flask was removed from the water bath and allowed to cool to room temperature. Subsequently, 20 mL of n-heptane was added with precision, and the mixture was shaken for 2 min, followed by the addition of a saturated aqueous sodium chloride solution to enable phase separation. The upper n-heptane layer, approximately 5 mL, was then pipetted into a 25 mL test tube, to which approximately 4 g of anhydrous sodium sulfate was added, shaken for 1 min, and allowed to stand for 5 min. The clarified upper layer was subsequently transferred into an injection vial for analysis [30]. The fatty acid methyl esters (FAMEs) were analyzed with an Agilent 6890 gas chromatograph (Operating conditions were as follows: column: HP-88 Agilent 100 m × 0.25 mm × 0.20 μm; detector: FID detector; detector temperature: 260 °C; inlet temperature: 250 °C; carrier gas: N2; flow rate: 1.0 mL/min; shunt injection, shunt ratio: 40:1) (Agilent Technologies, Inc., Santa Clara, CA, USA). The fatty acid composition in foot muscles was then determined by prevalent comparison with 37 FAME standards from Sigma (St. Louis, MO, USA).

### 2.5. RNA Extraction, Transcriptome Sequencing, and Data Processing

Total RNA was isolated from purified snail foot muscle tissue using a commercial Trizol kit (Invitrogen, Carlsbad, CA, USA) with instructions. After assessing the integrity and amount of total RNA extracted, mRNA was therefore enriched using Oligo(dT) magnetic beads and removing rRNA. The enriched mRNA was fragmented into short segments using divalent cations and subsequently reverse transcribed to synthesize the first strand of cDNA. The second strand of cDNA was synthesized using DNA polymerase I with dNTPs as the substrate. After quantitative purification, the double-stranded cDNA undergoes end repair, A-negative tailing, ligating with adapters, and PCR expansion to form the final library. Validation and quantification of library quality using the Agilent 2100 Bioanalyzer (Agilent Technologies, Inc., CA, USA) and qRT-PCR. The quantified library was then sequenced using the Illumina NovaSeq 6000 platform (Illumina, Inc., San Diego, CA, USA).

Raw data were filtered using Fastp (version 0.18.0) to eliminate reads containing adapters, undetermined bases (N), and low-quality reads (Qphred scores ≤ 20 for more than 50% of the bases). Following filtering, the clean data were assessed for Q30 and GC content. All subsequent high-quality analyses were based on clean data. Clean data were assembled using Trinity (version 2.1.1) to generate reference sequences, and the integrity and accuracy of the assembly were evaluated with BUSCO [31]. The resulting uniqueness were functionally annotated using established databases such as Nr, Nt, KEGG, COG, GO, and SwissProt. Differences in gene expression and metabolite profiles in different samples were examined using PCA. The DEGs between different groups were identified using DESeq2 software (version 3.0), applying a significance threshold of *p* < 0.05 and an absolute log2 fold change (FC) greater than 1 [32]. To illustrate the characteristics of these DEGs, the GO and KEGG databases were referenced for feature enrichment analysis. These analyses were performed using KOBAS 2.0 for KEGG and Goseq software (version 2.12) for GO.

### 2.6. Untargeted Metabolomic Profiling and Data Processing

Each muscle tissue sample, weighing approximately 100 mg, was pulverized under liquid nitrogen conditions. This homogenized tissue was then combined with 80% methanol, adjusted to a 53% final concentration, and centrifugated at 15,000× *g* for 20 min at a temperature of 4 °C to extract metabolites. The supernatant extracted was analyzed using Liquid Chromatography-Tandem Mass Spectrometry (LC-MS/MS) (Thermo Fisher Scientific Inc., Waltham, MA, USA) [33]. Metabolomic profiles were normalized and summarized according to established protocols. Only compounds from quality control (QC) samples with a relative standard deviation (RSD) of less than 30% were included, enhancing metabolite identification and relative quantification. These metabolites were then mapped to databases such as KEGG, HMDB, and LIPID MAPS for annotation. PCA was used to explore the differences between the samples. The metabolomic differences across various groups were examined through Orthogonal Projections to Latent Structures-Discriminant Analysis (OPLS-DA). Metabolites with Variable Importance in Projection (VIP) scores higher than 1 and *p*-values lower than 0.05 were categorized as discrepant metabolites.

### 2.7. Statistical Analysis

Data were analyzed using SPSS software (Version 22.0) (International Business Machines Corporation, Armonk, NY, USA). Differences in amino acid and fatty acid content within snail foot muscle among different groups were assessed by one-way analysis of variance (ANOVA). Prior to this, the normality and homogeneity of data distribution were tested by Shapiro–Wilk test and Levene’s test, respectively.

## 3. Results

### 3.1. Change in Growth Performance

After 80 d of culture, WG and SGR of *B. purificata* were significantly lower in the MD and HD groups than in the LD group (Table 1, *p* < 0.05), whereas the differences in WG and SGR between the MD and HD groups were not significant (Table 1, *p* > 0.05).

### 3.2. Amino Acids Concentrations

**Figure 1:**
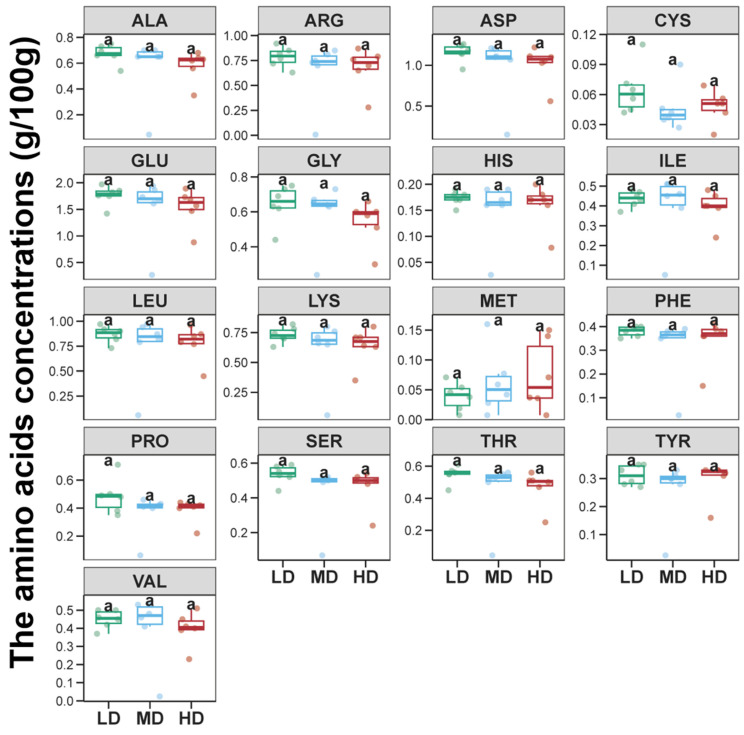
Differences in the alanine (Ala), arginine (Arg), asparagine (Asp), cystine (Cys), glutamic acid (Glu), glycine (Gly), histidine (His), isoleucine (Ile), leucine (Leu), lysine (Lys), methionine (Met), phenylalanine (Phe), proline (Pro), serine (Ser), threonine (Thr), tyrosine (Tyr), and valine (Val) within snail *Bellamya purificata* foot muscle between the three groups. Identical lowercase letters indicate that there is no significant difference between the three groups (*p* > 0.05).

A total of 17 amino acids were identified in the foot muscles of snails *B*. *purificata* (Figure 1). These included seven essential amino acids, two semi-essential amino acids, and eight non-essential amino acids (Figure 1). Among the essential amino acids, leucine had the highest content at over 0.75 g/100 g, followed by lysine at over 0.60 g/100 g (Figure 1). Among the non-essential amino acids, glutamic acid is the most abundant, exceeding 1.5 g/100 g, followed by aspartic acid at over 1.0 g/100 g (Figure 1). Cystine had the lowest content among the non-essential amino acids, with levels in the LD, MD, and HD groups all below 0.07 g/100 g (Figure 1). The results showed that there was no noticeable difference in amino acid content between the three groups (Figure 1, *p* > 0.05).

### 3.3. Fatty Acids Concentrations

**Figure 2:**
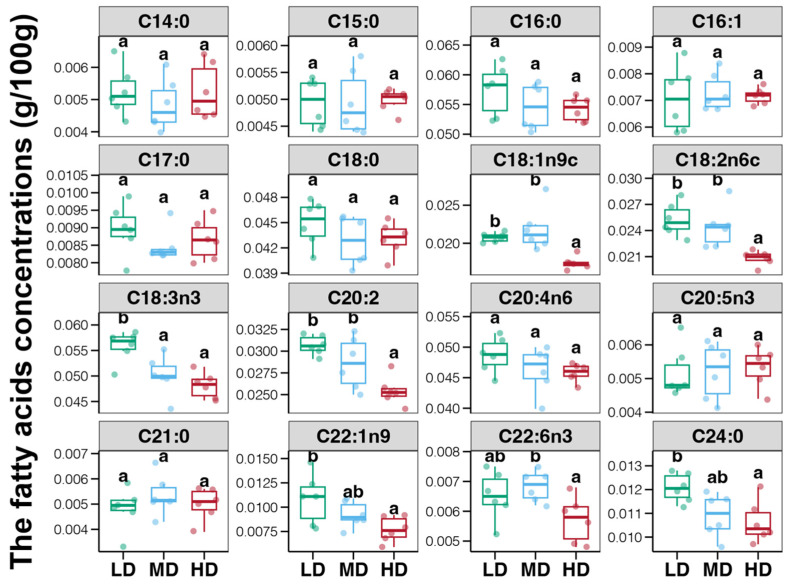
Differences in the myristic acid (C14:0), pentadecanoic acid (C15:0), palmitic acid (C16:0), palmitoleic acid (C16:1), margaric acid (C17:0), stearic acid (C18:0), cis-9-octadecenoic acid (C18:1n-9c), linoleic acid (C18:2n6c), alpha-linolenic acid (C18:3n3), eicosadienoic acid (C20:2), arachidonic acid (C20:4n6), eicosapentaenoic acid (EPA, C20:5n3), heneicosanoic acid (C21:0), erucic acid (C22:1n9), docosahexaenoic acid (DHA, C22:6n3), and lignoceric acid (C24:0) within snail *Bellamya purificata* foot muscle between the three groups. Different lowercase letters indicate significant differences between the three groups (*p* < 0.05).

There were in total sixteen fatty acids identified in the foot muscles of the snail *B*. *purificata*, including seven saturated (SFA), three monounsaturated (MUFA), and six polyunsaturated fatty acids (PUFA) (Figure 2). Palmitic acid (C16:0) was the predominant SFA, reaching levels above 0.055 g/100 g, followed by stearic acid (C18:0) (Figure 2). C18:3n3 was the most abundant PUFA, exceeding 0.050 g/100 g, with arachidonic acid (C20:4n6) next in concentration (Figure 2). The SFAs, except for C24:0, showed no obvious variances between the three groups (Figure 2, *p* > 0.05). The lignoceric acid concentration substantially decreased as stocking densities increased, reaching its lowest level in the high group (Figure 2, *p* < 0.05). Among the MUFAs, the concentration of C22:1n9 was significantly lower in the HD group than in the LD group (Figure 2). The concentration of C18:1n9c was also lower in the HD group than in LD and MD groups (Figure 2, *p* < 0.05). Meanwhile, among the PUFAs, C18:2n6c, C18:3n3, and C20:2 contents were evidently lower in the high-density group than in the low-density group (Figure 2, *p* < 0.05).

### 3.4. Transcriptome Sequencing and Analysis

After filtering the raw data, 19,318,506 to 22,963,651 clean reads were obtained (Table 2). The Q30 values ranged from 90.71% to 91.73% and the GC content ranged from 37.74% to 38.83%, all within the normal range, suitable for further analysis (Table 2). The minimum and maximum lengths of transcripts were 301 bp and 31,733 bp, respectively, which are the same as those of the unigenes (Table 3). The N50 values were 2407 for the transcripts and 1905 for the unigenes, while the N90 values were 576 and 437, respectively (Table 3).

**Figure 3:**
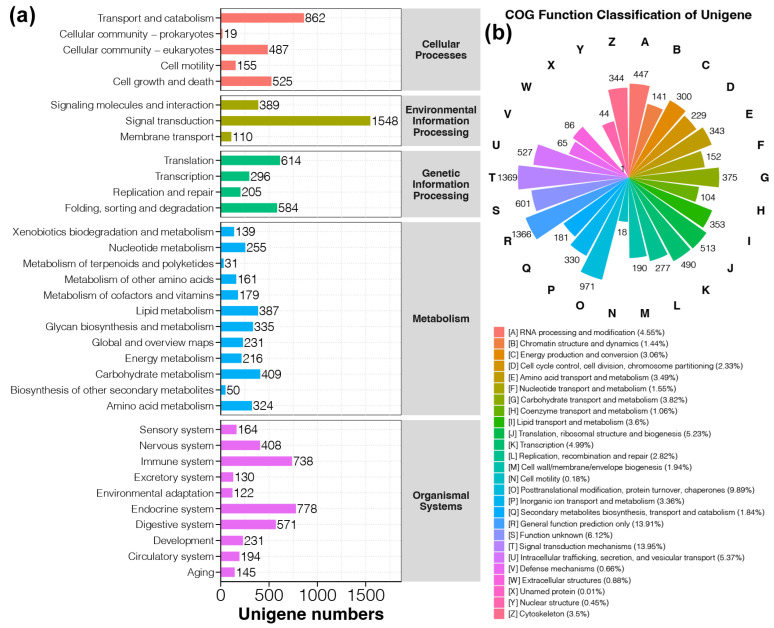
Functional annotations for the transcriptome sequencing in foot muscles of snail *Bellamya purificata*. (**a**) KEGG for annotation of snail *Bellamya purificata* foot muscle transcriptome sequencing. (**b**) COG annotations for the transcriptome sequencing across snails *B. purificata* foot muscles.

The genes identified in this study were mainly enriched in five major categories (Figure 3a). Among these, the Signal transduction in the Environmental Information Processing category had the most associated genes, with 1548 identified (Figure 3a). This was followed by the Transport and catabolism in the Cellular Processes category, with 862 associated genes identified (Figure 3a). COG functional annotation classified all the functional genes into 26 categories (Figure 3b). The top three categories by gene count were Signal transduction mechanisms, General function prediction only, and Posttranslational modification, protein turnover, and chaperones, with 1369, 1366, and 971 genes, respectively, representing 13.95%, 13.91%, and 9.89% of the total functional genes (Figure 3b).

**Figure 4:**
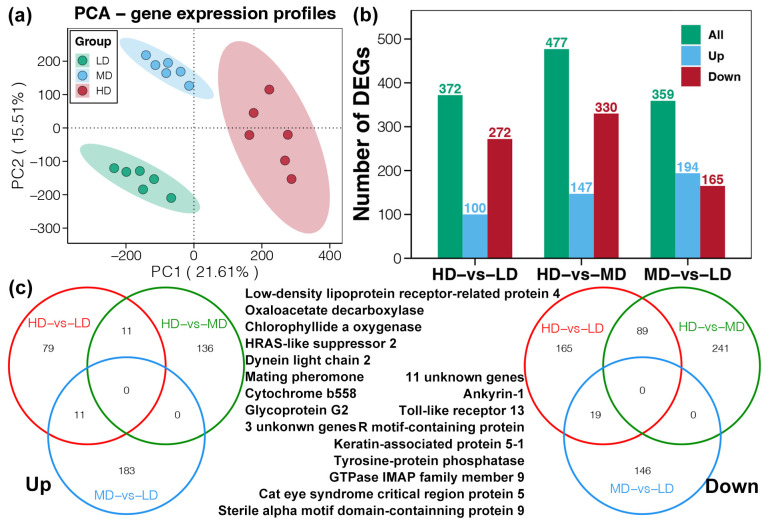
Differences in the functional gene expression within foot muscles of snail *Bellamya purificata* between the three groups. (**a**) PCA evaluating the differences in the gene expression profiles of snail foot muscle among the three groups. (**b**) Numbers of the DEGs in the three groups comparisons. (**c**) Venn diagram identifying the specific DEGs shared by three comparisons: HD vs. LD, HD vs. MD, and MD vs. LD.

The PCA results revealed that the LD, MD, and HD groups were remarkably clustered into three categories, which indicated that different stocking densities impacted the functional gene expression profiles in foot muscle of snail *B. purificata* (Figure 4a). DGE analysis identified 359 DEGs between the groups of LD and MD, of which 194 were upregulated and 165 downregulated (Figure 4b). In the comparison between the groups of LD and HD, 372 genes were differentially expressed, with 100 upregulated and 272 downregulated (Figure 4b).

Further analysis through Venn diagrams identified specific DEGs common to the three comparisons (Figure 4c). No specific DEGs were found across all three comparison groups (Figure 4c). The upregulated genes shared by the HD vs. LD and HD vs. MD comparisons were considered the principal genes affected by high density stress, numbering 11 genes (Figure 4c). Excluding three unknown genes, the functions of the other DEGs were as follows: Low-density lipoprotein receptor-related protein 4, Oxaloacetate decarboxylase, Chlorophyllide an oxygenase, HRAS-like suppressor 2, Dynein light chain 2, Mating pheromone, Cytochrome b558, and Glycoprotein G2 (Figure 4c). Similarly, the downregulated genes shared by the HD vs. LD and MD vs. LD comparisons were considered the principal genes affected by density stress, numbering 19 genes (Figure 4c). Excluding 11 unknown genes, the functions of the other DEGs were Ankyrin-1, Toll-like receptor 13, R motif-containing protein, Keratin-associated protein 5-1, Tyrosine-protein phosphatase, GTPase IMAP family member 9, Cat eye syndrome critical region protein 5, and Sterile alpha motif domain-containing protein 9 (Figure 4c).

### 3.5. Metabolomic Analysis

**Figure 5:**
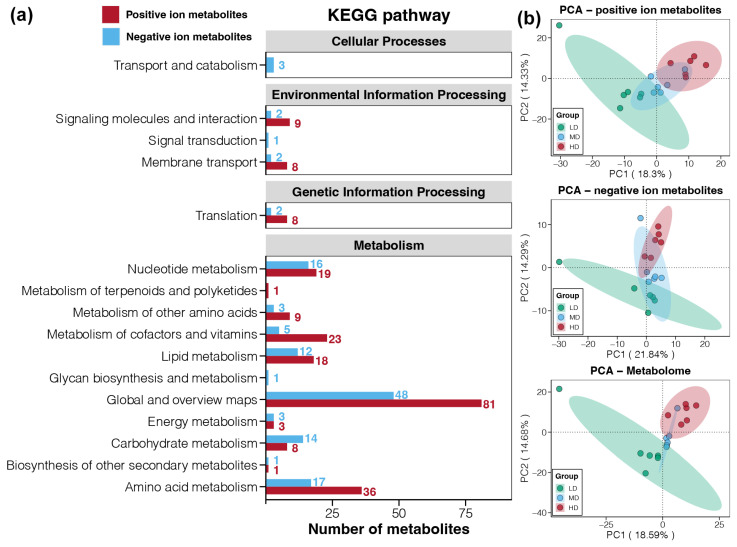
Functional annotations for the metabolites in foot muscles of snail *Bellamya purificata*. (**a**) KEGG annotations for the metabolites in foot muscles of snail *Bellamya purificata*. (**b**) PCA assessed the differences in the metabolomics of the snail foot muscles in three groups.

In the metabolomics of snail foot muscle, 885 metabolites were identified, including 620 in the POS and 265 in the NEG (Table 4). KEGG enrichment analysis identified three primary classifications in POS: Metabolism, Environmental Information Processing and Genetic Information Processing (Figure 5a). In NEG, there were four: Environmental Information Processing, Cellular Processes, Metabolism, and Genetic Information Processing (Figure 5a). Metabolism was particularly enriched with 81 and 48 Global and overview maps-related metabolites identified in POS and NEG, respectively (Figure 5a). In the Genetic Information Processing category, the Translation classification was noted, with eight metabolites annotated in POS and two in NEG (Figure 5a). In summary, most of the identified metabolites were amino acids, nucleic acids, vitamins, and lipid metabolites.

**Figure 6:**
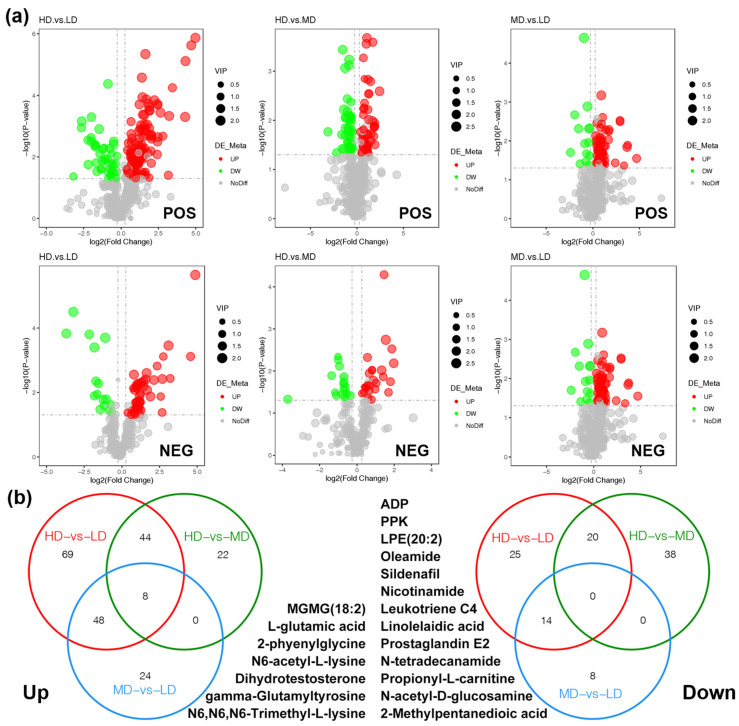
Differences in metabolites in foot muscles of snails from group LD, group MD and group HD. (**a**) The volcano plot for the differential metabolites under POS and NEG between the three groups. (**b**) Venn diagram identifying the specific differential metabolites shared by three comparisons: HD vs. LD, HD vs. MD, and MD vs. LD.

PCA results demonstrated distinct clustering of the LD and HD groups in both ion modes, indicating clear separation based on gene expression of the snails, while the MD group showed a high similarity to both LD and HD groups, highlighting significant impacts of density stress on gene expression (Figure 5b). Differential metabolite screening, using criteria of VIP > 1, |FC| > 1.2, and *p* < 0.05, identified significant variability: in POS mode, the HD vs. LD comparison revealed 167 different metabolites (124 upregulated, 43 downregulated); in NEG mode, there were 61 (45 upregulated, 16 downregulated) (Table 4 & Figure 6a). Subsequent Venn diagram analysis identified eight upregulated metabolites shared by the three comparisons (Figure 6b). No shared downregulated metabolites were identified across all the three comparisons. The 14 downregulated metabolites common between HD vs. LD and MD vs. LD, and the 20 downregulated metabolites shared by HD vs. LD and HD vs. MD, were considered the main metabolites affected by high density stress (Figure 6b).

## 4. Discussion

### 4.1. Effects of Stocking Density on Growth Performance

Studies have shown that under limited food and space resources, high-density aquaculture can intensify competition among aquatic animals, and in addition, high density can cause stress reactions in aquatic animals, change their intrinsic physiological conditions, and reduce growth rates and survival rates [34,35,36]. Tan et al. (2023) found that high-density stocking reduced the survival rate of *Crassostrea angulata* during the nursery stage [37]. Qi et al. (2016) investigated the effect of stocking density on growth performance of Juvenile blunt snout bream in a recirculating aquaculture system and found that higher stocking densities negatively affected the growth performance of individuals. Final body weight, specific growth rate (SGR), and weight gain decreased significantly with increasing stocking density [38]. Similarly, in the present study, after 80 days of culture, body weight, WG, and SGR were significantly lower in MD and HD groups than in LD group, indicating that high stocking density was not favorable for the growth of *B. purificata*. It is possible that high density induces a stress response in *B. purificata*, which in turn affects its growth efficiency [39].

### 4.2. Effects of Stocking Density on Amino Acid Composition in Snail Foot Muscle

Snail muscle is rich in nutrients and has a balanced amino acid profile, making it a high-quality protein source [9]. Stocking density is one of the key factors influencing muscle nutritional composition in aquatic animals, with excessively high density leading to a decline in muscle nutrients [25,26,40]. Protein content in muscle tissue is a crucial indicator of muscle quality. As the basic building blocks of proteins, amino acids can influence the composition and arrangement of muscle proteins, thereby affecting muscle texture and playing a crucial role in both muscle quality and flavor [41]. Wang et al. (2022) studied Chinese Mitten Crab in a pond ecosystem and found that the muscle tissue of Chinese Mitten Crab in the low density group contained higher levels of various amino acids such as leucine, lysine, essential amino acids, total amino acids, and umami amino acids compared to the high density group [26]. In contrast, Zhang et al. (2021) found that *Fenneropenaeus chinensis* did not show significant changes in free amino acids between groups of different culture densities in their study [42]. Similarly, for our study, there were also no significant changes in amino acids within snail foot muscle across different stocking densities. Wu et al. (2000) found that under starvation, the Chinese shrimp preferentially utilizes lipids and proteins for its energy requirements [43]. It is possible that the order of energy metabolizing substance species differed under different stress conditions, while under high-density stress snail *B. purificata* preferentially utilized carbohydrates and lipids as energy sources [42].

### 4.3. Effects of Stocking Density on Fatty Acid Composition in Snail Foot Muscle

The fatty acid composition of muscle is an indicator of an organism’s nutritional status and health [44]. Arachidonic acid, DHA, and EPA are critical long-chain highly unsaturated fatty acids (LC-HUFAs) that regulate growth performance and antioxidant status for the farmed aquatic animals [45,46]. Some investigations have shown that high-density farming affects the crude fat content of fish muscle [47]. Liu et al. (2019) observed that the PUFA, MUFA, and SFA concentrations in plasma were significantly decreased in juvenile Lenok (*Brachymystax lenok*) raised under a high-density condition [48]. Similarly, in this study, the C18:1n9c, C18:2n6c, C18:3n3, C20:2, C22:1n9, C22:6n3, and C24:0 levels within snail foot muscle under the high stocking density condition were significantly lower than the middle or low-density stocking condition. A large number of studies have confirmed that stressful conditions lead to metabolic alterations [49]. The substantial reductions in fatty acids were likely due to stress from high-density conditions affecting metabolic processes, thereby altering the fatty acid composition of snail foot muscle. These findings might reveal that high-density farming has adverse effects on the nutritional status and health of foot muscle in snail *B. purificata*.

### 4.4. Effects of Stocking Density on Snail Foot Muscle Metabolic Function

In the aquaculture industry, high stocking densities induce chronic stress that detrimentally impacts animal welfare and negatively influences the biosynthesis and metabolism of lipids, proteins, and carbohydrates in organisms [40,50]. Lipids, serving as the primary source of metabolic energy, are particularly vulnerable, as inappropriate densities can destabilize the steady state of lipid metabolism in fish species [51]. Previous research indicated that high stocking density in grass carp culture could lead to abnormalities in lipid and carbohydrate metabolism and impair immune function by affecting the expression levels of genes encoding Major Histocompatibility Complex (MHC) molecules [52]. Similarly, research on the metabolic responses of *Brachymystax lenok* has demonstrated that elevated farming densities can modify amino acid and choline metabolism and suppress lipid metabolism [48]. For this study, the metabolomic analysis revealed that the identified metabolites were enriched in the Metabolism category, while the differential metabolites affected by stocking density were primarily amino acids, nucleic acids, vitamins, and lipid metabolites. Among them, high culture density caused downregulation of Linolelaidic acid, N-Acetyl-D-glucosamine, and Nicotinamide metabolites. Linolelaidic acid and N-acetyl-D-glucosamine are metabolites with anti-infective and anti-inflammatory properties [53,54]. Two metabolites were downregulated, suggesting that high-density culture has a detrimental effect on the anti-inflammatory properties of the organisms. Nicotinamide promotes lipid synthesis and alleviates oxidative stress and inflammatory responses [24]. Nicotinamide metabolites were downregulated in the high-density group, indicating that lipid metabolism and antioxidant status were negatively affected. In addition, high stocking densities caused upregulation of L-glutamic acid metabolites, indicating enhanced neurotransmission in the organism [55]. This may be an adaptive response mechanism for the organism to cope with high density stress.

Meanwhile, in this study, Venn diagrams highlighted the downregulation of the Toll-like receptor 13 and the Sterile alpha motif domain-containing protein 9 genes. The Toll-like receptor family plays a pivotal role in mediating innate immunity, serving as the first defense line against diseases in aquatic animals and bridging innate and adaptive immune responses. Toll-like receptor 13, a member of this family, is integral to immune and inflammatory responses [56]. The Sterile alpha motif domain-containing protein 9 genes play a key role in the innate immune response to stimuli such as viral infection [57]. The observed downregulation of TLR13 and SAMD9 might suggest that high stocking density adversely affected the organism’s innate immune capabilities. Mating pheromone, which is critical to reproductive behaviors across various organisms and promotes attraction and mating, was found to have its gene upregulated by high stocking density [58,59]. Additionally, the differential metabolite dihydrotestosterone (DHT) upregulated in this study, is a testosterone-derived androgen and crucial for developing male characteristics such as body hair [60]. Abnormal levels of DHT can lead to complications like the underdevelopment of external genitalia, prostate enlargement, and hair loss [60]. Casalini, M. et al. (2010) on *Rhodeus ocellatus* found that high density greatly affected the proportion of males defending mussel spawning grounds and the proportion of mussels releasing sperm and had a significant effect on male courtship rates [61]. Weir, L.K. (2013) found that high male densities in *Oryzias latipes* cause intense competition during mating, which may have led to the evolution of alternative mating strategies to improve the success of male fertilization [62]. In this study, the upregulation of the mating pheromone and the metabolite DHT under high stocking density, suggesting a density-dependent stress response. These upregulations might promote competitive mating behaviors among individuals, facilitating the selection of mates with superior genetic qualities and resources [61,63,64].

## 5. Conclusions

In summary, high-intensity aquaculture adversely affects the muscles of snail *B. purificata*. The growth parameters of snail *B. purificata* were reduced at culture densities above 234.38 g/m^2^. In addition, high stocking density significantly reduced the C18:1n9c, C18:2n6c, C18:3n3, C20:2, C22:1n9, C22:6n3, and C24:0 levels within snail foot muscle. PCA results highlighted significant impacts of high-density stress on gene expression and metabolite profiles in snail foot muscle. Most of the identified metabolites were categorized as Signal transduction, Transport and catabolism, Endocrine system, and Immune system according to KEGG. A total of 11 upregulated DEGs and 19 downregulated DEGs were identified and confirmed to be associated with density stress. The identified metabolites were mainly enriched in Metabolism category, with 620 differential metabolites identified in POS mode and 265 differential metabolites were identified in NEG mode among different stocking density groups. The differential metabolites affected by stocking density were primarily amino acids, nucleic acids, vitamins, and lipid metabolites. There were 8 upregulated differential metabolites and 14 downregulated differential metabolites identified and confirmed to be associated with density stress. The observed changes in these metabolites may indicate an adaptive response of *B. purificata* to unfavorable conditions. These findings demonstrated the adverse effects of a high-density condition on the nutritional quality and metabolic functions of snail muscle and provide some data and theoretical basis for selecting appropriate stocking densities for *B. purificata*. Considering all the growth performance and foot muscle nutritional quality and metabolic function, the optimal stocking density of *B. purificata* was recommended to be around 234.38 g/m^2^.

## Figures and Tables

**Table 1:** Growth parameters of snail *Bellamya purificata* at different stocking densities.

Groups	W1 (g)	W2 (g)	WG (%)	SGR (%/d)
LD	2.53 ± 0.01	3.00 ± 0.05 ^a^	18.35 ± 2.21 ^a^	0.21 ± 0.02 ^a^
MD	2.53 ± 0.01	2.79 ± 0.06 ^b^	10.91± 2.58 ^b^	0.13 ± 0.03 ^b^
HD	2.53 ± 0.01	2.80 ± 0.03 ^b^	10.65± 0.37 ^b^	0.13 ± 0.01 ^b^

Note: The same column of data with different letters indicates that the difference is significant (*p* < 0.05). Values are presented as means ± SD (*n* = 3).

**Table 2:** Statistics of transcriptomics sequencing dataset.

Sample ID	Raw Read Numbers	Clean Read Numbers	Clean Bases (Gb)	Error Rate (%)	Q30 (%)	GC (%)
LD_1	20,446,237	20,019,811	6.0	0.03	90.71	38.55
LD_2	19,617,041	19,318,506	5.8	0.03	91.27	38.21
LD_3	20,650,117	20,313,130	6.1	0.03	91.06	38.1
MD_1	22,641,805	22,208,129	6.7	0.03	91.12	38.19
MD_2	23,440,617	22,963,651	6.9	0.03	91.42	38.4
MD_3	21,770,598	21,308,818	6.4	0.03	91.73	38.39
HD_1	22,985,521	22,542,846	6.8	0.03	91.17	37.74
HD_2	20,255,282	19,981,273	6.0	0.03	91.36	37.87
HD_3	23,103,528	22,787,013	6.8	0.03	91.25	38.83

**Table 3:** Statistics of transcript assembly.

Type	Min Length (bp)	Mean Length (bp)	Median Length (bp)	Max Length (bp)	N50 (bp)	N90 (bp)	Total Nucleotides (bp)
Transcript	301	1452	849	31,733	2407	576	496,976,958
Unigene	301	1134	598	31,733	1905	437	160,828,491

**Table 4:** Differential metabolites screening in the three comparisons.

Compared Samples	Num. of Total Ident.	Num. of Total Sig.	Num. of Sig. Up	Num. of Sig. Down
MD vs. LD (POS)	620	81	67	14
HD vs. LD (POS)	620	167	124	43
HD vs. MD (POS)	620	91	49	42
MD vs. LD (NEG)	265	21	13	8
HD vs. LD (NEG)	265	61	45	16
HD vs. MD (NEG)	265	41	25	16

## Data Availability

Transcriptome-related data supporting this manuscript have been uploaded to the National Center for Biotechnology Information (NCBI) database with the accession number PRJNA1175440.

## References

[1] Jin W., Cao X.J., Ma X.Y., Lv G.H., Xu G.C., Xu P., Sun B., Xu D.P., Wen H.B. (2022). Chromosome-level genome assembly of the freshwater *snail Bellamya purificata* (Caenogastropoda). Zool. Res..

[2] Hou Y.R., Li B., Luo J.W., Zhang C.F., He J., Zhu J. (2021). Effect of *Bellamya purificata* on organic matter degradation in surface sediment as revealed by amino acids. Aquac. Environ. Interact..

[3] Hou Y., Zhou M., Jia R., Sun W., Yang Y., Huang X., Li B., Zhu J. (2023). Effects of Snail *Bellamya purificata* Farming at Different Stocking Densities on the Algal and Fungal Communities in Sediment. Fishes.

[4] Jiao D.X., Xin X.C., Jiao L.Y., Nian Z., Feng Z., Jiao L.R. (2013). Feeding habits of *Bellamya purificata* and its function in water purification system of ecological ditch. Fish. Mod..

[5] Hao Z.M., Yun Z.Y., Ran H.Y., Bing L., Cheng F.G., Yu C.K., Jian Z. (2021). Effect of the Bioturbation Derived from Snail *Bellamya purificata* on Pond Sediments Nitrogen Forms. Trans. Oceanol. Limnol..

[6] Zhi Z.Z., Yuan M.X. (2024). Liuzhou River snails rice noodle industry boosting rural revitalization development. Agric. Eng. Technol..

[7] He M.B., Hua W.H., Wu J., Bin H., Huan L.Y., Hong L.Z., Jie W.M., Gang L.C. (2022). Research on the water purification effects of the combination of snail *Bellamya purificata* and alage *Elodea Nuttallii*. Jiangxi Fish. Sci. Technol..

[8] Zhang Y., Hou Y., Jia R., Li B., Zhu J., Ge X. (2022). Nitrogen occurrence forms and bacterial community in sediment influenced by *Bellamya purificata* bioturbation. Front. Mar. Sci..

[9] Hui L., Ting C.L., Sen J.T., Bo S.W., Zhe L., Rui Z.M., Qi Q.J., Song D.X., Ting W.L., Hui P.X. (2022). Muscle nutrition analysis of four snail species of Viviparidae. J. Fish. China.

[10] Jie Y.Y., Wu J., Bo W.H., Yan M.X., Ting X., Fei S.C., Jin H.Y., Wen B.X. (2018). Estimation of genetic parameters for growth traits of *Bellamya purificata* in 60 days. Freshw. Fish..

[11] Jun Y., Xue D.L., Hong W.Y., Jie H., Guang L.F., Ming J. (2023). The Impact of Feed Fat Levels on the Nutritional Components and Growth Performance of *snail Bellamya Purificata*. Sci. Fish Farming.

[12] Yun G.Y., Xin G.S., Bo P. (2024). The rapid development of shellfish mariculture and the continuous optimization and upgrading of the entire industry chain. China Food.

[13] Moniruzzaman M., Uddin K.B., Basak S., Mahmud Y., Zaher M., Bai S.C. (2015). Effects of Stocking Density on Growth, Body Composition, Yield and Economic Returns of Monosex Tilapia (*Oreochromis niloticus* L.) under Cage Culture System in Kaptai Lake of Bangladesh. J. Aquac. Res. Dev..

[14] Xiu D.L., Yong L.J., Xiang P., Qian Y., Qiang X., Zhi D., Hui G. (2023). Effects of Stocking Density on Rice Yield, Rice Quality and Ecological Environment in the Coculture of Rice and Aquatic (poultry) Animals. China Rice.

[15] Chowdhury M.A., Roy N.C., Chowdhury A. (2020). Growth, yield and economic returns of striped catfish (*Pangasianodon hypophthalmus*) at different stocking densities under floodplain cage culture system. Egypt. J. Aquat. Res..

[16] Liu B., Fei F., Li X., Wang X., Huang B. (2019). Effects of stocking density on stress response, innate immune parameters, and welfare of turbot (*Scophthalmus maximus*). Aquac. Int..

[17] Merino G.E., Piedrahita R.H., Conklin D.E. (2007). The effect of fish stocking density on the growth of California halibut (*Paralichthys californicus*) juveniles. Aquaculture.

[18] Refaey M.M., Li D., Tian X., Zhang Z., Zhang X., Li L., Tang R. (2018). High stocking density alters growth performance, blood biochemistry, intestinal histology, and muscle quality of channel catfish Ictalurus punctatus. Aquaculture.

[19] Pires-Júnior A.N., Hattori G.Y., Sant’Anna B.S. (2019). Effect of stock density of cultured Amazon Apple Snail Pomacea dolioides (*Gastropoda: Ampullariidae*) in Brazil. Rev. Bras. Zootec..

[20] Ling L.Z., Hua S.Y., Xing Z.X., Feng G.Z. (2023). Effects of different culture densities on the intermediate cultivation of *Babylonia areolate* in a recirculating aquaculture system. J. Trop. Biol..

[21] Ying P., Ping W.X., Jin C.D., Chao P.J., Zhen H.S., Xun W., Yu C., Kang L., Jian C., Jia X.X. (2012). Studies on reared in suspended-cage of Hemifusus Tuba (Gmelin). Trans. Oceanol. Limnol..

[22] Yi W.T., Peng R.L., Yan Z.C. (2024). Effect of density on the nonspecific immunity of Scylla serrata. North. Chin. Fish..

[23] Lu C., Yan Y.O., Muhammad R., Ding Y.D., Muhammad K.I., Feng Y., Juan Z., Shuang H.J. (2021). Compensatory growth in gibel carp (*carassius auratus gibelio*) after the stress of stocking density. Aquac. Res..

[24] Hang D.C., Rui J., Ran H.Y., Bing L., Jian Z. (2023). Effects of stocking density on the antioxidant capacity, muscle nutrient composition and metabolism function of Micropterus salmoides in integrated rice-bass farming systems. J. Fish. Sci. China.

[25] Meng N., Mei L., Feng L.J., Qiang M.G., Lin Y.J., Min G.Z. (2021). Stocking density alters growth performance, serum biochemistry, digestive enzymes, immune response, and muscle quality of largemouth bass (*Micropterus salmoides*) in in-pond raceway system. Fish Physiol. Biochem..

[26] Yu W.Z., Fang M.Z., Yong L.X., Jie Z.M., Jie L.L., Feng M., Ying H.P., Hao H.J., Qin J.S., Xian Z.D. (2022). Growth Performance, Nutritional Quality, and Immune-Related Gene Expression of the Chinese Mitten Crab (*Eriocheir sinensis*) in Pond Ecosystem as Influenced by Stocking Density. Fishes.

[27] Di G.K., Hua R.Y., Ying F.H., Ru Z.Y. (2023). Effects of High-Fat Diets on Growth, Liver Fat Deposition, Fatty Acid Composition and Intestinal Microorganisms of Grass Carp (*Ctenopharyngodon idellus*). Henan Fish..

[28] Feng Z., Xin X.C., Nian Z., Jiao L.Y., Jiao L.R. (2014). Efficiency of Water Purification and the Nitrogen and Phosphorous Release of the Sediment by Different Densities of *Bellamya purificata*. J. Hydroecol..

[29] Jiao D.X. (2013). Feeding Habits and Selectivity of *Bellamya purificata* in Ecolofical Ditch. Master’s Thesis.

[30] (2016). Determination of Fatty Acids in Foods.

[31] Grabherr M.G., Haas B.J., Yassour M., Levin J.Z., Thompson D.A., Amit I., Adiconis X., Fan L., Raychowdhury R., Zeng Q. (2011). Full-length transcriptome assembly from RNA-Seq data without a reference genome. Nat. Biotechnol..

[32] Zhang Z.H., Jhaveri D.J., Marshall V.M., Bauer D.C., Edson J., Narayanan R.K., Robinson G.J., Lundberg A.E., Bartlett P.F., Wray N.R. (2014). A comparative study of techniques for differential expression analysis on RNA-Seq data. PLoS ONE.

[33] Want E.J., Masson P., Michopoulos F., Wilson I.D., Theodoridis G., Plumb R.S., Shockcor J., Loftus N., Holmes E., Nicholson J.K. (2013). Global metabolic profiling of animal and human tissues via UPLC-MS. Nat. Protoc..

[34] Domínguez-Godino J.A., González-Wangüemert M. (2018). Does space matter? Optimizing stocking density of *Holothuria arguinensis* and *Holothuria mammata*. Aquac. Res..

[35] Damodaran D., Koya K.M., Mojjada S.K., Lalaji C.D., Dash G., Vase V.K., Sreenath K.R. (2018). Optimization of the stocking parameters for mud spiny lobster *Panulirus polyphagus* (Herbst, 1793) capture-based aquaculture in tropical open sea floating net cages. Aquac. Res..

[36] Cubillo A.M., Peteiro L.G., Fernández-Reiriz M.J., Labarta U. (2012). Influence of stocking density on growth of mussels (*Mytilus galloprovincialis*) in suspended culture. Aquaculture.

[37] Tan K.R., Zhai Y.T., Zhang H.K., Zeng Z.A., Ning Y., Zheng H.P. (2023). Effects of culture conditions (stocking density, water depth and aquaculture gear) on the aquaculture performance of a new Crassostrea angulata variety “Golden oyster#1”. Aquaculture.

[38] Qi C.L., Xie C.X., Tang R., Qin X., Wang D.Y., Li D.P. (2016). Effect of Stocking Density on Growth, Physiological Responses, and Body Composition of Juvenile Blunt Snout Bream, *Megalobrama amblycephala*. J. World Aquac. Soc..

[39] Shao T.Y., Chen X.Y., Zhai D.X., Wang T., Long X.H., Liu Z.P. (2019). Evaluation of the effects of different stocking densities on growth and stress responses of juvenile hybrid grouper ♀ *Epinephelus fuscoguttatus* × ♂ *Epinephelus lanceolatus* in recirculating aquaculture systems. J. Fish Biol..

[40] Zhao H., Fan O.S., Xia J., Tang R., Li L., Li D. (2019). Transcriptome and physiological analysis reveal alterations in muscle metabolisms and immune responses of grass carp ( *Ctenopharyngodon idellus*) cultured at different stocking densities. Aquaculture.

[41] Xiao Z.W., Qing J.Q., Lei X.S., Yan T.H., Fei L., Ping Y.W., Bing Y.Y., Gang Y.Z., Qiang X.Z., Zhi Z.Y. (2021). Research Progress on the Impact of Amino Acids on Muscle Quality in Aquatic Animals. Prog. Fish. Sci..

[42] En Z.H., Ying H.Y., Jian L., Xu H., Jun X.Y. (2021). Effects of Different Stocking Densities on the Growth and Energy Metabolism of *Fenneropenaeus chinensis*. Prog. Fish. Sci..

[43] Wu L.X., Dong S.L., Wang F., Tian X.L. (2000). Compensatory growth response following periods of starvation in Chinese shrimp, *Penaeus chinensis* Osbeck. J. Shellfish Res..

[44] Yun D.L., Ping Z.Y., Cheng C.J., Jing C.W., Qi X.S., Tang C.Q. (2022). Growth, Muscle Nutrition Composition, and Digestive Enzyme Activities of the Juvenile and Adult Siniperca chuatsi Fed on Live Baits and a Formulated Diet. Fishes.

[45] Zhan F.S., Zheng C., Yu X., Kun L.H., Dong H., Yan J.J., Xia Y.Y., Ming Z.X., Qi X.S. (2020). Effects of dietary arachidonic acid on reproduction performance, tissue fatty acid profile and gonadal steroidogenesis in female yellow catfish Pelteobagrus fulvidraco. Aquac. Nutr..

[46] Wu J., Xiong W., Liu W., Wu J., Ruan R., Fu P., Wang Y., Liu Y., Leng X., Li P. (2024). The Effects of Dietary n-3 Highly Unsaturated Fatty Acids on Growth, Antioxidant Capacity, Immunity, and Oxylipin Profiles in *Acipenser dabryanus*. Antioxidants.

[47] Yang Q., Guo L., Liu B.S., Guo H.Y., Zhu K.C., Zhang N., Jiang S.G., Zhang D.-C. (2020). Effects of stocking density on the growth performance, serum biochemistry, muscle composition and HSP70 gene expression of juvenile golden pompano Trachinotus ovatus (Linnaeus, 1758). Aquaculture.

[48] Liu Y., Liu H., Wu W., Yin J., Mou Z., Hao F. (2019). Effects of stocking density on growth performance and metabolism of juvenile Lenok (*Brachymystax lenok*). Aquaculture.

[49] Rong Y., Li B., Hou Y., Zhang L., Jia R., Zhu J. (2024). Influences of Stocking Density on Antioxidant Status, Nutrients Composition, and Lipid Metabolism in the Muscles of *Cyprinus carpio* under Rice-Fish Co-Culture. Antioxidants.

[50] Hematyar N., Rahimnejad S., Waghmare S.G., Malinovskyi O., Policar T. (2024). Effects of Stocking Density and Pre-Slaughter Handling on the Fillet Quality of Largemouth Bass (*Micropterus salmoides*): Implications for Fish Welfare. Foods.

[51] Yousefi-Garakouei M., Kamali A., Soltani M. (2019). Effects of rearing density on growth, fatty acid profile and bioremediation ability of polychaete Nereis diversicolor in an integrated aquaculture system with rainbow trout (*Oncorhynchus mykiss*). Aquac. Res..

[52] Yan H., Yan Y.H., Gang Z.H., Hua Z., Jing Z.Q., Qi W.A., Bang S.Y., Yan X.X., Le L.J. (2021). Transcriptomic analysis to elucidate the effects of high stocking density on grass carp (*Ctenopharyngodon idella*). BMC Genom..

[53] Azuma K., Osaki T., Minami S., Okamoto Y. (2015). Anticancer and Anti-Inflammatory Properties of Chitin and Chitosan Oligosaccharides. J. Funct. Biomater..

[54] Li J., Rao H., Bin Q., Fan Y.W., Li H.Y., Deng Z.Y. (2017). Linolelaidic acid induces apoptosis, cell cycle arrest and inflammation stronger than elaidic acid in human umbilical vein endothelial cells through lipid rafts. Eur. J. Lipid Sci. Technol..

[55] Doltchinkova V., Mouleshkova N., Vitkova V. (2021). Surface Properties of Synaptosomes in the Presence of L-Glutamic and Kainic Acids: In Vitro Alteration of the ATPase and Acetylcholinesterase Activities. Membranes.

[56] Xiuqin T. (2022). Cloning of TLR13 Gene from *Nibea albiflora* and Exploration of Related Immune Mechanism. Master’s Thesis.

[57] de Matos A.L., Liu J., McFadden G., Esteves P.J. (2013). Evolution and divergence of the mammalian SAMD9/SAMD9L gene family. Bmc Evol. Biol..

[58] Wei G.J., Ying W.W., Ming Y.X., Long W.S., Bo L.T., Feng L.Y., Hong Z., Shuang G.Q., Jun D.Y. (2024). Calling and mating behaviors of *Sesamia inferens* (*Lepidoptera: Noctuidae*) moths in the environment of high does sex pheromone for mating disruption. Acta Entomol. Sin..

[59] Kobayashi M., Sugiura M., Iwasaki S., Iwabe N., Harumoto T. (2024). Mating Pheromone (Gamone 1) inBlepharisma: A Glycoprotein Responsible for Species Diversity in Unicellular Organisms (*Alveolata, Ciliophora*). Microorganisms.

[60] Bhawna C., Priyanka S., Umesh R., Brototi R. (2023). Exploring the effect of dihydrotestosterone on NOD-like receptors expression in spotted snakehead Channa punctata (Bloch 1793). J. Fish Biol..

[61] Casalini M., Reichard M., Smith C. (2010). The effect of crowding and density on male mating behaviour in the rose bitterling (*Rhodeus ocellatus*). Behaviour.

[62] Weir L.K. (2013). Male-male competition and alternative male mating tactics influence female behavior and fertility in Japanese medaka (*Oryzias latipes*). Behav. Ecol. Sociobiol..

[63] Holwell G.I., Allen P.J.D., Goudie F., Duckett P.E., Painting C.J. (2016). Male density influences mate searching speed and copulation duration in millipedes (*Polydesmida: Gigantowales chisholmi*). Behav. Ecol. Sociobiol..

[64] Lin D.X., Ying Z.M., Bo J., Qian G.Z., Fei J., Jian S., Jiang D.F. (2016). Effects of stocking density on growth, sexual differentiation and gonad development of Macrobrachium rosenbergii. J. Fish. China.

